# Race, class, and marriage: Components of race differences in men’s first marriage rates, United States, 1960–2019

**DOI:** 10.4054/demres.2022.46.39

**Published:** 2022-06-29

**Authors:** Steven Ruggles

**Affiliations:** 1 Minnesota Population Center, University of Minnesota, Minneapolis, Minnesota, USA.

## Abstract

**BACKGROUND:**

Wilson (1987) argued that race differences in the frequency of marriage from the 1960s to the 1980s resulted from a shortage of marriageable men in the Black community. A large literature used spatially defined measures of male marriageability to predict marriage rates of women. These studies concluded that the availability of marriageable men can explain only a fraction of race differences in marriage. I argue that this finding may reflect errors in the measurement of the availability of marriageable spouses.

**METHODS:**

My analysis assesses marriage rates from the perspective of men instead of women, allowing direct assessment of men’s economic positions without resorting to fuzzy spatial indicators. I develop new measures of first marriage rates for the 1960–1980 censuses and combine them with survey-based measures for 2008–2019. I use classic decomposition methods to assess the changing relationship of economic composition to race differences in male first marriage rates over the 1960–2019 period.

**RESULTS:**

The analysis shows that in the mid- to late 20^th^ century, race differences in economic composition were sufficiently large to account for most race differences in first marriage rates, but the size of the economic component declined dramatically over time.

**CONCLUSIONS:**

With the decline of male-breadwinner families since the late 20^th^ century, the role of male economic circumstances for race differences in marriage rates has diminished, but it remains substantial.

**CONTRIBUTION:**

Leveraging a new research strategy and a new measure of first marriage, this analysis provides a consistent decomposition of race differences in first marriage rates over six decades.

## 1. Introduction

Black marriage rates have been lower than White marriage rates for the past 60 years. Wilson (1987) argued that lower marriage rates in the Black community resulted from a shortage of employed men relative to the number of unmarried women. To quantify the mismatch, Wilson and Neckerman (1986) proposed the Male Marriageability Pool Index (MMPI), which they defined as the number of employed men in a marriage market per 100 women. They showed that MMPI ratios fell sharply in the 1970s and early 1980s for Blacks below age 35 as Black marriage rates declined and the Black–White marriage gap grew.

The usual strategy for evaluating the Wilson hypothesis is to develop measures of the availability of marriageable men across geographic areas – defined as neighborhoods, cities, labor market areas, metropolitan areas, or states – and to use those measures in a model predicting the marital status of women. Marriageability is sometimes determined purely by employment, but most analyses also account for earnings and other indicators of economic well-being (e.g., Lichter et al. 1992; South and Lloyd 1992; Fossett and Kiecolt 1992; Testa and Krough 1995; Wood 1995). Some studies use longitudinal data to assess the impact of male economic circumstances on the race differential in the incidence of marriage (Bloome and Ang 2020; Lichter et al. 1992; Oppenheimer Kalmijn, and Lim 1997; Sweeney 2002; Testa and Krogh 1995).

Virtually all these studies found that the availability of marriageable men had a significant impact on marriage rates of women (Cragie, Darity, and Myers 2018). At the same time, the literature found that only a fraction of the marriage differential between Whites and Blacks can be ascribed to differences in the availability of marriageable men (e.g., Brien 1997; Cohen and Pepin 2018; Darity, Myers, and Bowman 1995; Lichter et al. 1992; Lichter, LeClere, and Mclaughlin 1991; Lloyd and South 1996; McLaughlin and Lichter 1997; Raley 1996; Wood 1995). Most of the race difference in marriage of women remains even after controlling for the economic characteristics of their potential spouses. The emerging consensus is that “something more than class status is at play” in the low marriage rates of Blacks compared with Whites (Raley, Sweeney, and Wondra 2015: 100). In sum, the studies stimulated by Wilson’s marriageability thesis demonstrated that men’s employment and earnings are significantly associated with marriage rates, but they also argue that this effect is insufficient to explain most of the observed race differences in marriage.

The fuzziness of spatially defined measures of marriageability could be responsible for the small effects found in the literature. The spatial approach used in the bulk of the literature on race differences in marriage is designed to capture the relationship of the socioeconomic status of potential husbands to the incidence or prevalence of marriage among women. This strategy has significant limitations. It is impossible to identify the universe of potential spouses for any particular woman. The pool of potential spouses for any particular individual depends on their social networks, which may be defined by characteristics such as socioeconomic status, education level, church membership, kin relationships, and school or work contacts. Potential spouses are not defined by any particular spatial unit, such as metropolitan area or labor market area. For example, the availability of unmarried suburban professional men may have little impact on the marriage prospects of unmarried mothers with limited education residing in a central city, even though they reside in the same metropolitan area. Accordingly, spatially defined measures of the availability of potential spouses are inherently noisy and cannot fully capture the actual marriage opportunities available to any particular person.

This problem is compounded by substantial measurement error in the availability of employed unmarried men. Some classic studies of the 1990s relied on the 1% Public Use Microdata Sample-D (PUMS-D) of the 1980 census to estimate the characteristics of potential husbands, because it provided information on labor market areas (e.g., Lichter, LeClere, and McLauglin 1991; Lichter et al. 1992; Lloyd and South 1996). On average, labor market areas in the D Sample included just 52 unmarried Black men aged 20 to 39, so the analyses were limited to the unrepresentative subset of labor market areas with a high frequency of Black men. Brien (1997) used the larger 5% A Sample from 1980 and compared the effects of male characteristics on female marriage incidence measured at three levels of spatial aggregation: county groups, metropolitan areas, and states. He found that male characteristics measured at the state level could account for most of the race differences in marriage incidence, whereas male characteristics measured at the level of county groups or metropolitan areas had a much smaller effect. Brien attributed this finding to measurement error in the sub-state measures. State populations vastly overbound the universe of potential partners; Brien’s findings underscore that spatially defined measures do not realistically describe the actual pool of potential husbands.

We can sidestep the limitations of contextual measures by measuring marriage rates from the perspective of men rather than women. Wilson 1987 was primarily based on observations from 1960 to 1980, so this is the critical period for evaluating the hypothesis. In this era, marriages in the United States were universally monogamous and heterosexual, and marriages between Blacks and Whites were still rare. Therefore the incidence of male first marriages was virtually identical to the incidence of female first marriages among both Blacks and Whites, and it makes little technical difference whether we measure marriage rates from the perspective of women or men.

Wilson argues that the race differential in marriage resulted from race differences in the marriageability of men, which in turn was tied to male economic circumstances and incarceration. If this is true, we would expect little difference in marriage rates between Black and White men who shared the same degree of marriageability. To test Wilson’s hypothesis, we can directly measure the individual-level relationship between male marriageability and marriage rates. The hypotheses focuses on the characteristics of men, so it makes sense to evaluate it by looking at the demographic behavior of men. It is far more straightforward to conduct this analysis for men than for women because we do not need to rely on noisy and unreliable inferences about a pool of potential spouses; instead, we can directly measure male marriageability at the individual level.

A second major advantage of analyzing the relationship of men’s economic characteristics to their own marriage behavior is that we can for the first time assess long-run changes in the race difference in marriage. None of the previous analyses allow analysis of long-run change because the available spatial indicators are inconsistent across time. My study encompasses both the 1960–1980 period – the period to which Wilson’s hypothesis was originally applied – and the more recent period from 2008 to 2019. Because of the dramatic increase in women’s workforce participation – especially among married women – it is reasonable to anticipate that marriage rates in the 21^st^ century would be less sensitive to male circumstances than they were a half century earlier.

## 2. Measures and data

### 2.1 Measuring first marriage rates

Past census-based studies of race differences in marriage focus on the prevalence of marriage, such as the percentage of women ever married in a particular age group, such as age 22 to 27 (Fitch and Ruggles 2000). Such measures are not suitable for an individual-level analysis of the association between marriage and economic position because of significant mismatch between the timing of marriage and the observation of economic characteristics. If we focus on the percent ever married, the marriage can occur years prior to the observation of economic circumstances. By instead measuring the incidence of first marriage, we can ensure that the economic measures are approximately contemporaneous with the marriages.

This analysis is based on a new measure of marriage rates in the decennial censuses of 1960, 1970, and 1980 together with a comparable measure from the American Community Surveys (ACS) of 2008–2010 and 2017–2019. First marriage rates are defined as the number of persons marrying for the first time during a 12-month period per thousand persons who had never been married at the start of the period. This measure is appropriate for assessing the relationship of economic circumstances to marriage, since the available economic measures are approximately contemporaneous with marriage.

We can construct first marriage rates for the 1960, 1970, and 1980 census microdata by combining responses to four variables: age, age at first marriage, birth quarter, and marriage quarter. All persons whose current age is the same as their age at first marriage married within the past 12 months, since we know that they had a birthday within 12 months of the census, and if the marriage had occurred prior to the birthday, the marriage age would not match the current age.

In addition, persons whose age at first marriage is one year less than their current age married within the past year if their marriage date preceded their birth date. Consider, for example, a man listed in the 1960 census at age 26 who was married at age 25. The census reference date was April 1, 1960, so we need to determine whether he was married between April 1959 and March 1960. Assume he married in May and turned 26 in October. We can infer that he was 25 in May 1959 and therefore married within the prior year. Conversely, if his 26^th^ birthday was in May 1959 and his marriage date was in October, we can infer that the marriage took place in October 1958 and that he was not married in the 12 months preceding the census.

Unfortunately, the census does not give exact dates of birth and marriage. Rather, we have information on only quarter of marriage and quarter of birth. In most cases, this allows us to establish whether the marriage preceded the birthday, but if the marriage quarter is the same as the birth quarter, it does not reveal which event came first. In these cases, we can turn to spousal information. The great majority of newlyweds reside with their spouses, and in cases of uncertainty we can use a spouse’s information to resolve ambiguity. Assuming the spouse was also getting married for the first time, her marriage within a year applies to her husband as well.

By combining information on both spouses, it is possible to unambiguously determine whether the marriage occurred within the past year in approximately 90% of cases. For the remaining 10% of cases, I assume that the marriage preceded the birthday half the time. Accordingly, I randomly assigned marriage to 50% of the remaining ambiguous cases. The rules for constructing first marriage rates from the census are summarized in Table 1.

To evaluate the plausibility of marriage rates derived from the census, I compared them with age-specific marriage rates derived from vital records for 1960, 1970, and 1980. These are available only for persons in the Marriage-Registration Area (MRA), which was established in 1957 by the National Office of Vital Statistics to provide detailed statistics on marriage – including age at marriage and previous marital status – for a subset of states that agreed to cooperate. In 1960 the system was still being developed; it covered just 62% of first marriages, and analysts expressed concern about underreporting of marriages and about the high percentage of characteristics “not stated” (National Center for Health Statistics 1964). By 1970, 90% of first marriages were covered by the MRA system.

**Figure 1:**
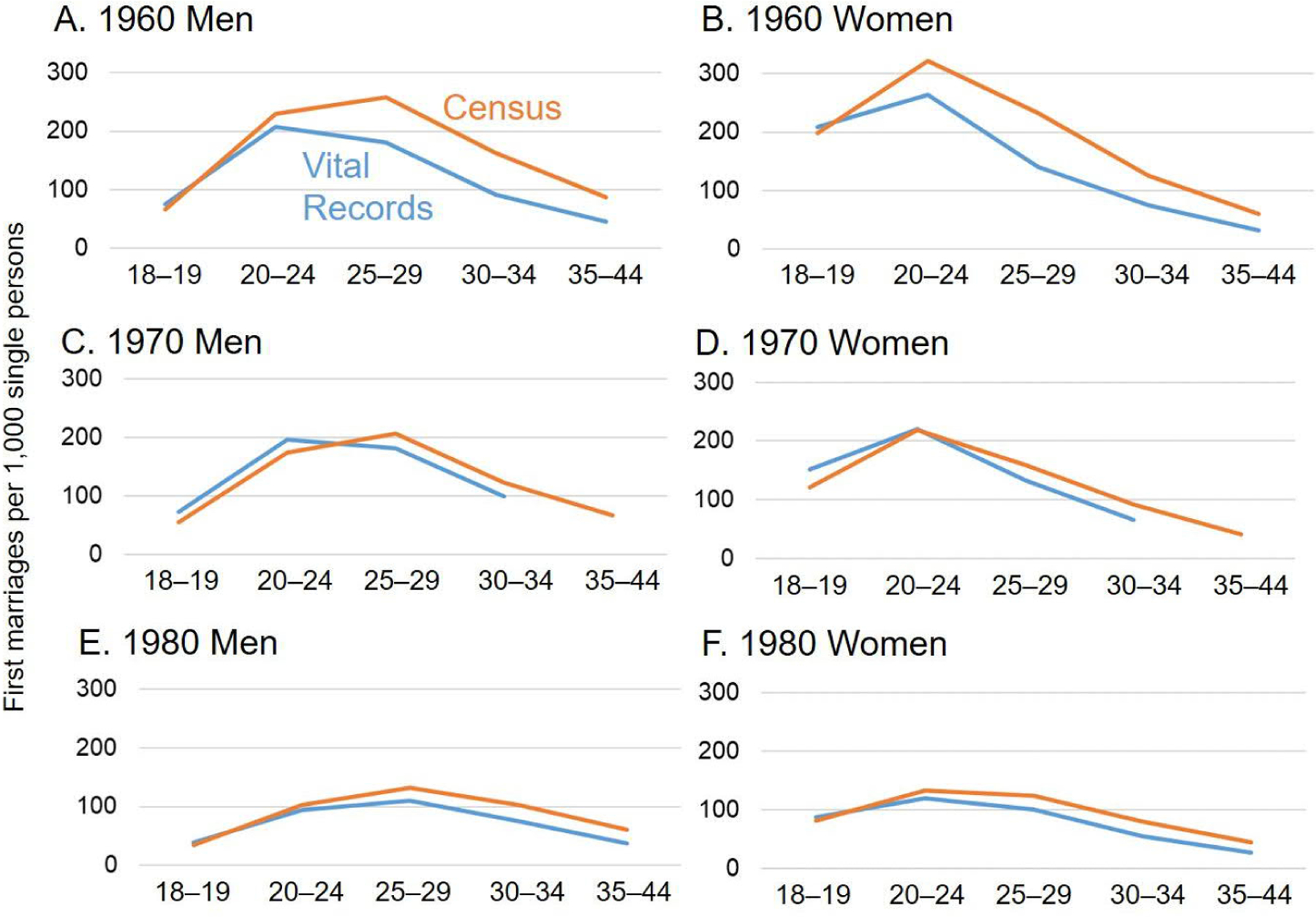
Comparison of age-specific first marriage rates from the census and vital records: Marriage-Registration Area, 1960–1980 *Sources*: National Center for Health Statistics 1964, 1974, 1985 and Ruggles et al. (2021).

Figure 1 compares age-specific first marriage rates for the MRA derived from census microdata with those derived from vital records. Cases with allocated values for age at first marriage or number of times married are excluded. In most cases, the age-specific first marriage rates derived from the census reasonably approximate the rates calculated from marriage certificates, but there are some exceptions. Most notably, in 1960 the rates derived from the census for persons aged 25 or older are somewhat higher than the rates from vital statistics.

It is unclear whether differences between the two sources reflect errors in the census or errors in vital statistics. The census might overstate marriage rates at older ages if respondents misreported remarriages as first marriages, although there is no obvious reason to suspect that this problem would be worse in 1960 than in later years. In 1960 vital statistics likely had some underreporting of first marriages under the newly formed MRA system. Moreover, the vital registration system did not count marriages conducted without a legal marriage certificate, and such marriages may have been more common in 1960 than in later census years. The convergence between estimates from the census and from vital records between 1960 and 1980 may reflect improvements in the quality of both sources.

It is straightforward to construct a directly comparable measure from recent American Community Surveys. In 2008 the ACS added two questions that make it simple to calculate first marriage rates: “(1) In the PAST 12 MONTHS did this person get married? (2) How many times has this person been married?” (Elliott, Simmons, and Lewis 2010). I calculate the first marriage rate as the number of persons married once and married within the past 12 months per thousand people who had never married at the beginning of the 12-month period.

The National Center for Health Statistics discontinued the collection of detailed marriage statistics from vital records in 1996, so it is not possible to use the same approach used for the censuses to evaluate the quality of marriage rates derived from the ACS. However, the Census Bureau did compare crude marriage rates from the ACS to rates estimated from vital records, and it concluded that the two sources yield similar results (Elliott, Simmons, and Lewis 2010).

### 2.2 Data sources and study population

In 1990 the census dropped the question on age of first marriage. Accordingly, this analysis is limited to the earlier period (1960 through 1980) along with the period since 2008, when marriage questions were added to the ACS. The study population consists of US-born non-Hispanic men aged 18–49 who are identified in the census as Black or White. The censuses of 1960 through 1980 report a single race for each respondent. The ACS allows for multiple-race responses; I used the Black alone and White alone categories. I also tested Black alone or in combination with other races and White alone or in combination. The results were virtually unaffected, probably because multiple-race responses are still comparatively infrequent.

I use 11 samples from IPUMS (Ruggles et al. 2021). For the earlier period, I use the new 5% sample of the 1960 census, the three 1% Form 1 samples of the 1970 census, and the 5% state sample of the 1980 census. For the ACS, I combine three years of microdata to obtain sufficient cases for the decomposition analysis, comparing 2008–2010 (the earliest three-year period available) and 2017–2019 (the most recent three-year period available).^2^

**Figure 2:**
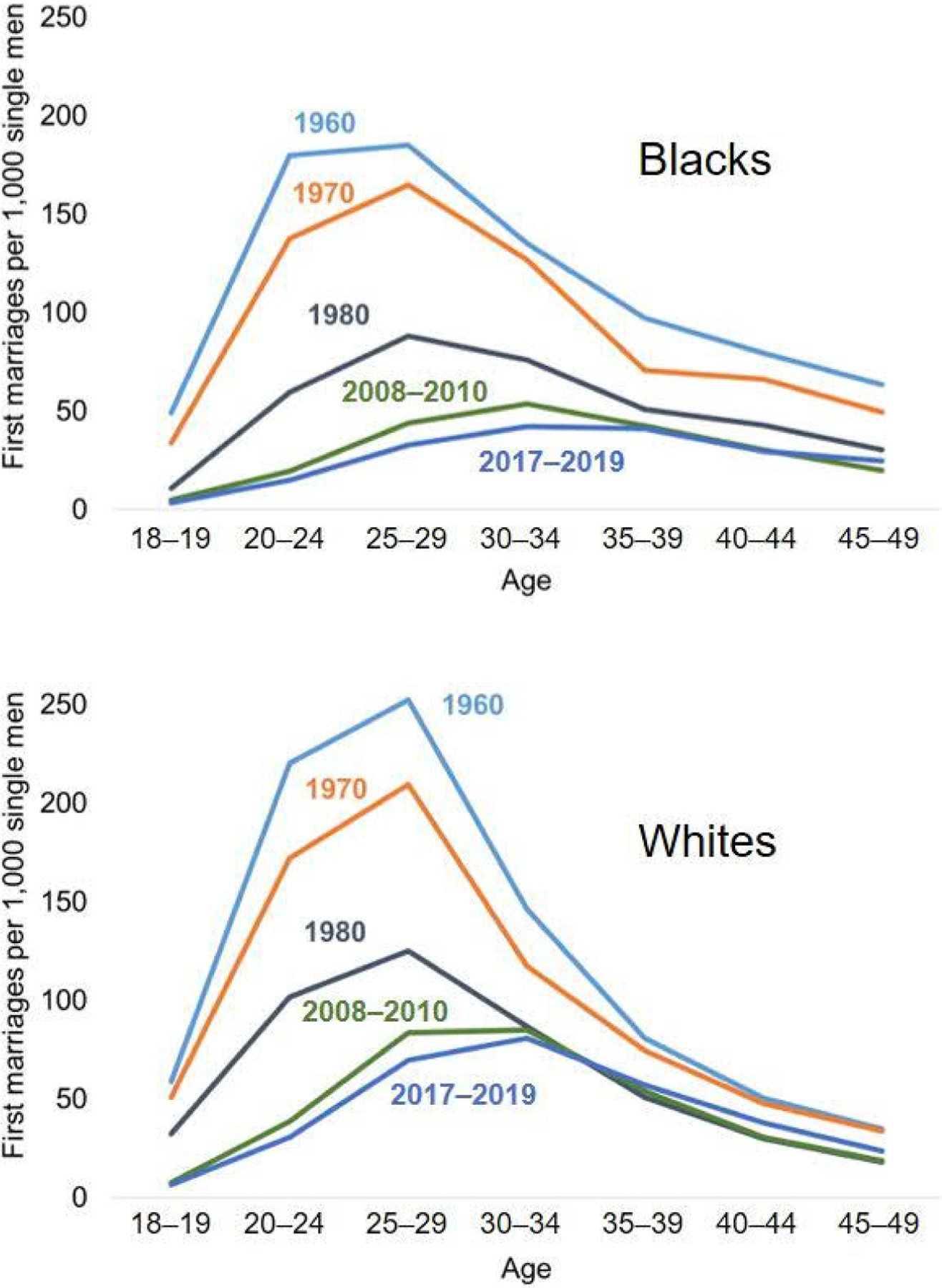
Age-specific first marriage rates by census year: US-born non-Hispanic Black and White men *Source*: Ruggles et al. 2021.

The age-specific first marriage rates for each race and period appear in Figure 2. At every age in every period, Black men married substantially less frequently than did White men. Among both Blacks and Whites, marriage rates declined precipitously between 1960 and 2017–2019, especially among young adults.

### 2.3 Measures of marriageability

To assess how much of the race differential in marriage rates between 1960 and 2019 can be attributed to differing circumstances, I measure race differences in population composition with respect to several economic characteristics. I treat marriageability as a continuum rather than a dichotomy. As Wood (1995) noted, marriage is more sensitive to income level than to employment, so studies that focus exclusively on the availability of employed men for marriage may understate the impact of economic circumstances on marriage. Moreover, combinations of socioeconomic characteristics – such as income, job type, employment, and incarceration – have greater explanatory power than any single characteristic.

Table 2 describes the three economic factors included in the analysis: income, occupational group, and employment/institutionalization.

- Total individual income is divided as closely as possible into deciles calculated separately for each period.^3^ In all periods, Black men are overrepresented in the bottom decile and underrepresented in the upper deciles.
- Occupation is divided into four groups. Professional, managerial, and skilled workers (1950 census codes 0–299 and 500–599) are predominately White in all census years; clerical workers, salespeople, and operatives (codes 300–499 and 600–699) are mixed; and service workers and laborers (codes 700–899) are overrepresented among Blacks. The IPUMS variable YRLASTWK is used to make the “no occupation” category consistently mean “no occupation within the past five years.”
- Employment/institutionalization combines information from the IPUMS variables EMPSTAT, WORKEDYR, and GQ to make a consistent variable covering both employment status and institutionalization. Incarceration has had a major impact on race differences in marriage rates (Charles and Luoh 2020). Although the data to not permit consistent identification of the incarcerated population, we can identify the institutionalized. Whites are overrepresented among the currently employed, and Blacks are overrepresented among persons who have not worked in the past year or who reside in institutions.

Although these three measures overlap, they also capture subtly different aspects of employment rather than Wilson’s simple dichotomy. Because my analysis applies classic decomposition methods and does not use regression, collinearity is not a concern. Each factor makes a substantial net contribution to the composition component of the race difference in marriage rates.^4^

First marriage rates for each factor appear in Table 3. Each of the factors has a profound relationship to marriage rates for both Black men and White men. Men in the top income decile married between five and ten times as frequently as men in the bottom decile. Employment and occupation also have a striking association with first marriage rates. The overall race difference in first marriage rates ranges from 15.6 per thousand in 1960 to 28.0 per thousand in 1980. Compared with the massive difference in first marriage rates between economic subgroups and across time, the differences between Black men and White men are comparatively small.

The powerful relationship between economic position and marriage rates is not necessarily purely the consequence of marriageability; other factors likely come into play. Most important, high-income men may be able to marry more frequently than low-income men because they can better afford to support a family. Moreover, men who plan to marry soon might strive to obtain a better-paying job, so the prospect of marriage could itself contribute to the positive association between economic position and first marriage rates. Because the available evidence does not address causes of the powerful association between economic status and first marriage, it is insufficient to support causal analysis. I therefore do not formally address the causes of the observed association between marriage and economic position. Instead, I assess whether the differences in the economic composition of the two populations are sufficiently large to account for the observed differences in first marriage rates. The appropriate tool to address this question is components analysis.

## 3. Analysis

Following Kitagawa (1955), we can divide the difference between two rates into a composition component and a rate component. The composition component is the part of the difference attributable to differences in population composition. The rate component is the difference between the populations that would remain even if their population compositions were identical. In addition to the three marriageability measures defined above, it is important to control for age, so my analysis includes four factors. The rate component with four factors can be calculated as

$$\text{Rate}\;\text{component}=\underset{i}{\sum }\underset{j}{\sum }\underset{k}{\sum }\underset{l}{\sum }\left(R_{\text{ijkl}}-r_{\text{ijkl}}\right)P_{\text{ijkl}}$$

where *R*_*ijkl*_ is the marriage rate for persons with characteristics i, j, k, and l in the first population being compared, *r*_*ijkl*_ is the corresponding rate for the other population, and *P*_*ijkl*_ is the proportion of the standard population with characteristics i, j, k, and l. The composition component is the difference between the two populations that remains once the rate component is removed.

This provides a simple approach for evaluating the relationship of differing economic compositions of the Black and White populations to first marriage rates, controlling for all interactions and avoiding assumptions of additivity or linearity. The analysis is based on cross classifications of rates and population distributions of the four independent variables with time and race, creating a matrix of 19,200 cells.

The choice of the standard population can have a significant impact on the result. We can ask two distinct questions:

1. What is the race difference in marriage if Whites are weighted to match the economic circumstances of Blacks?
2. What is the race difference in marriage if Blacks are weighted to match the economic circumstances of Whites?

Panel A of Table 4 shows the composition component and the rate component in each period, using the Black population as the standard.^5^ The left panel of the table shows the race difference expressed as first marriages per 1,000 never-married people, and the right panel shows the same information expressed as a percentage of the total race difference. The composition component shows how much of the race difference disappears if the White population is weighted to have economic characteristics and age compositions identical to those of the Black population.

In 1960 and 1970, the composition component is substantially greater than the total population difference in first marriage rates. In 1960, the composition component is over three times the actual population difference, and in 1970 it is 1.5 times as great. This means that Whites with the same economic circumstances as Blacks married much less frequently than did Blacks. The role of economic composition diminished over time but can still potentially account for more than 90% of the race difference in 1980 and a majority of the race difference in both periods since 2008.

Panel B is the same as Panel A, except that Whites are the standard population. The composition component shows how much of the race difference would disappear if Blacks had the same distribution of economic circumstances and age compositions as Whites. The general patterns are the same as in Panel A, but the composition component is consistently smaller. Moreover, if we use Whites as the standard, the composition component shrinks even more rapidly over time, falling below 50% in 2008–2010 and down to just 28.3% in 2017–2019.

The reason the two standards differ is that race differentials in marriage rates are generally smaller for men at the lower end of the economic spectrum than for those at the higher end. As can be seen in Table 3, this pattern became more pronounced over time. In the early period, low-income and unemployed Black men actually married more frequently than did Whites facing the same circumstances. In the more recent period, Black first marriage rates were lower than those of Whites in almost every economic category, but the disparity is especially large among the most marriageable groups.

**Figure 3:**
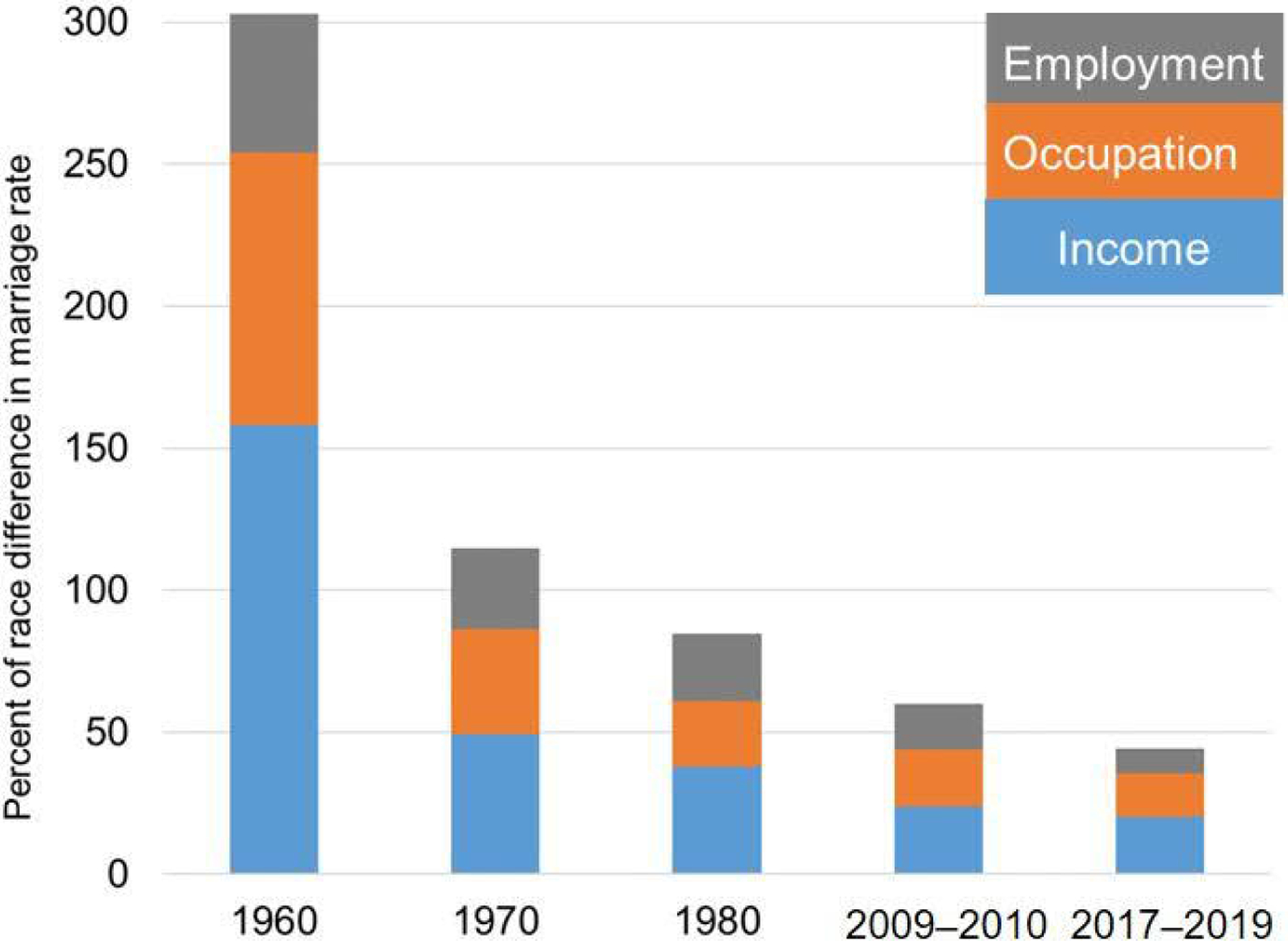
Economic components of the race difference in first marriage rates as a percentage of the total race difference in first marriage rates: US-born non-Hispanic Black and White men

We can subdivide the composition component into the four individual factors (income, occupation, employment/institutionalization, and age) following Das Gupta’s (1978, 1992) refinement of Kitagawa’s approach. For this analysis, the standard population is the average of the Black and White populations. Table 5 allocates the composition component into each of the three economic factors and age. Interactions between factors are distributed in proportion to the main components. Panel A is expressed in absolute first marriage rates, Panel B is expressed as a percentage of the total race difference in marriage rates, and Panel C shows each factor as a percentage of the composition component. The changing contributions of the three economic factors as a percentage of the total race difference in marriage rates are displayed graphically in Figure 3.

In all periods, income decile is the most important factor by a wide margin, on average contributing almost half of the composition component. Occupational category is the second most important factor, accounting on average for almost a third of the composition component. Employment status and institutionalization – the measure closest to Wilson’s original marriageability index – is ironically the weakest of the economic factors. The effect of age is generally negative, because in most periods the Black population is slightly younger than the White population and more highly represented in the peak ages for first marriage.

## 4. Discussion

Previous analyses of Wilson’s marriageability hypothesis have found that the association between men’s economic characteristics and marriage rates is insufficient to account for the race gap in marriage rates. This finding, I argue, is compromised by two sources of measurement error. First, the universe of women’s potential partners cannot be accurately described by assessing the economic characteristics of unmarried men within a particular geographic footprint; the actual pool of potential partners is defined by social networks that are much more difficult to measure. Second, as demonstrated by Brien (1997), available indicators of men’s marriageability for geographic units below the state level are unreliable for Black men, at least partly because of inadequate sample sizes.

This study minimizes measurement errors common to prior studies by measuring economic position of men directly at the individual level. I implement a new historical census-based measure of marriage rates that avoids the chronological mismatch in conventional cross-sectional measures of marriage. These innovations allow the first consistent analysis across six decades of changes in the economic component of race differences in first marriage rates.

Compared with White men, Black men have had disproportionately low income, low-skilled jobs, low employment, and high levels of incarceration. These differences are sufficiently large to account for most of the disproportionately low marriage rates of Black men in all but the most recent period. This analysis strongly supports Wilson’s central argument. As Wilson stressed, declining opportunities in manufacturing had devastating consequences for young Black men’s earnings and employment, and this was compounded by mass incarceration. During the period Wilson examined – the 1960s through the 1980s – Black men with economic and demographic characteristics similar to those of White men, measured by income, employment, and incarceration, had similar or higher marriage rates.

The potential effect of male economic circumstances on race differences in first marriage rates has waned in the 21^st^ century. With the decline of the male-breadwinner family, the relative importance of male economic circumstances on marriage prospects has diminished (Ruggles 2015, 2016). Women’s economic resources play an increasingly important role in marriage decisions, and higher earnings for women are now associated with higher marriage rates (Kuo and Raley 2014; Sweeney 2016). Women with independent resources face less pressure to marry, and some choose to delay marriage or forgo it entirely.

This analysis probably understates the importance of material circumstances for marriage prospects, both because it does not account for any of the circumstances of women and because it does not account for all the circumstances of men. The analysis includes institutionalization but not the impact of a prison record, which is much more common among Blacks than among Whites. Job insecurity is doubtless greater for Blacks than for Whites, but the components analysis cannot capture that. We have information about current income and occupation but nothing about the perceived future prospects of workers, which must on average be less bright for young Blacks than for young Whites. The census also has no information about wealth (or perceived prospects of future wealth), and wealth is substantially lower among Blacks than among Whites. Nor can we measure parental resources and debt, which have been shown to powerfully influence marriage rates (Bloome and Ang 2020).

Because of these omissions, the true effect of material circumstances on the race differential in first marriage is probably even greater than this analysis suggests. Were it possible to statistically control for all economic influences on the marriageability of men, we would likely find it sufficient to account for observed race differences in first marriage rates even in the most recent period.

## Figures and Tables

**Table 1:** Rules for assigning marriage within year in the 1960, 1970, and 1980 censuses

AGE: Age at last birthday
AGEMARR: Age at first marriage
BIRTHQTR: Quarter of birth
MARRQTR: Quarter of marriage
Persons are married within the past year if:
a) AGEMARR = AGE
b) AGEMARR = AGE–1 and MARRQTR precedes BIRTHQTR Census day is April 1, the first day of Quarter 2, so the marriage preceded the birthday if any of the following conditions are met:
MARRQTR = 2 and BIRTHQTR = 3, 4, or 1
MARRQTR = 3 and BIRTHQTR = 4 or 1
MARRQTR = 4 and BIRTHQTR = 1
If AGEMARR = AGE–1 and MARRQTR = BIRTHQTR, the case is ambiguous
c) Search for spouse and apply rules a and b to spouse’s information
d) If the case is still ambiguous, randomly assign first marriage to 50% of cases

**Table 2:** Percentage distribution of selected characteristics: Never-married non-Hispanic US-born Black men and White men, 1960–2019

	1960		1970		1980		2008–2010		2017–2019	
	Black	White	Black	White	Black	White	Black	White	Black	White
Decile of Income										
1	21.0	10.7	21.2	9.6	23.5	7.6	27.1	10.9	22.2	11.6
2	12.7	7.5	11.9	8.9	13.4	9.8	6.3	5.6	6.6	5.8
3	11.1	9.8	10.4	10.5	9.8	9.6	9.5	10.6	9.7	10.3
4	11.7	11.3	7.9	11.0	8.6	10.2	10.3	9.4	11.4	9.5
5	8.3	8.8	7.5	9.4	8.9	10.2	9.5	10.1	10.5	9.8
6	11.0	9.7	9.5	9.9	9.9	10.4	9.3	10.0	10.5	10.3
7	9.7	10.4	9.8	9.7	9.6	11.6	8.5	10.6	9.3	9.9
8	6.9	10.1	11.4	11.6	5.3	8.9	7.8	10.6	8.3	10.7
9	5.0	10.8	6.6	9.0	6.1	10.8	6.6	10.9	6.5	10.5
10	2.6	10.9	3.9	10.3	4.8	10.9	5.0	11.3	5.0	11.5
Employment and institutionalization										
Currently employed	60.2	72.1	55.4	66.9	51.0	70.7	47.8	68.1	58.2	72.8
Not employed but worked in past year	14.7	15.8	16.7	20.6	17.7	18.6	11.8	14.3	9.0	9.6
No work in past year	18.0	9.4	20.9	10.4	25.5	9.1	29.3	15.7	23.9	15.6
institutional residence	7.1	2.8	7.0	2.2	5.8	1.6	11.1	2.0	8.9	1.9
Occupation										
Professional, managerial, skilled	14.2	35.6	22.0	37.8	23.0	39.2	19.6	38.2	21.0	39.3
Clerical, sales, operative	21.9	32.7	31.1	32.8	28.7	31.2	27.8	27.0	28.7	25.6
Service, labor	37.5	17.2	29.2	21.7	29.4	23.8	28.0	24.8	27.2	23.5
No occupation in past five years	26.4	14.5	17.7	7.7	18.9	5.9	24.5	10.0	23.1	11.7
Total	100.0	100.0	100.0	100.0	100.0	100.0	100.0	100.0	100.0	100.0
Number of cases (unweighted)	389,724	54,153	295,113	42,315	683,114	121,767	481,550	108,274	538,531	115,523

*Note*: Includes men marrying within 12 months of census date.

**Table 3:** First marriages per thousand never-married population by selected characteristics: Never-married non-Hispanic US-born Black men and White men, 1960–2019

	1960		1970		1980		2008–2010		2017–2019	
	Black	White	Black	White	Black	White	Black	White	Black	White
Decile of Income										
1	41.6	32.1	35.2	32.2	22.7	27.1	8.5	11.9	7.4	10.0
2	71.8	48.8	57.4	40.2	33.4	33.0	11.5	10.4	10.1	10.8
3	115.7	63.0	62.1	55.1	39.4	35.2	14.7	13.6	15.8	14.4
4	151.7	103.3	86.7	74.5	50.7	49.5	17.1	19.5	12.7	18.2
5	202.7	150.6	114.3	105.4	69.3	70.9	21.3	31.1	18.9	26.6
6	231.4	197.4	141.2	144.2	102.8	103.5	37.1	44.5	28.0	41.4
7	261.2	244.3	176.7	189.4	115.9	130.4	47.3	59.8	34.3	55.3
8	251.4	271.4	202.5	227.2	118.4	147.1	54.5	76.2	43.7	71.4
9	255.3	278.3	226.8	242.2	131.3	163.0	61.3	91.2	47.8	89.2
10	237.1	254.5	194.9	208.1	134.7	155.9	65.0	95.4	59.5	104.3
Employment and institutionalization										
Currently employed	207.9	208.7	164.9	173.8	99.6	116.7	42.6	62.1	32.6	58.5
Not employed but worked in past year	106.8	85.1	63.5	59.1	43.8	48.5	24.3	23.5	13.4	18.8
No work in past year	46.5	34.9	37.0	37.6	23.4	26.5	8.6	12.3	7.7	10.8
institutional residence	43.8	28.9	39.1	28.5	32.4	29.0	13.3	16.9	12.0	8.5
Occupation										
Professional, managerial, skilled	205.1	208.3	145.1	176.8	102.1	122.0	50.0	72.1	42.5	70.4
Clerical, sales, operative	214.4	191.5	149.0	141.1	80.7	98.4	31.4	43.9	24.7	41.3
Service, labor	149.4	121.4	101.9	84.8	56.7	64.1	25.1	31.2	20.8	30.4
No occupation in past five years	76.8	71.6	25.2	18.6	16.2	12.6	6.9	7.2	6.3	8.1
Total	152.3	168.0	112.5	132.9	66.4	94.4	27.2	47.9	23.1	46.3

**Table 4:** Components of race difference in first marriage rates

A. Standard population = Blacks						
Year	Components of race difference			Components as % of race difference		
	Composition component	Rate component	Total difference	Composition component	Rate component	Total difference
1960	48.7	−33.0	15.8	308.8	−209.0	100.0
1970	30.0	−9.4	20.6	145.8	−45.6	100.0
1980	25.6	2.5	28.1	91.2	8.9	100.0
2008–2010	14.3	6.3	20.6	69.4	30.6	100.0
2017–2019	12.5	10.6	23.2	53.9	45.7	100.0
B. Standard population = Whites						
Year	Components of race difference			Components as % of race difference		
	Composition component	Rate component	Total difference	Composition component	Rate component	Total difference
1960	44.6	−28.8	15.8	282.3	−182.3	100.0
1970	24.1	−3.5	20.6	117.0	−16.9	100.0
1980	19.5	8.6	28.1	69.4	30.7	100.0
2008–2010	9.5	11.1	20.6	46.1	53.9	100.0
2017–2019	6.6	16.6	23.2	28.3	71.7	100.0

**Table 5:** Effect of individual factors on the race difference in first marriage rates

A. Absolute difference in marriage rates					
	1960	1970	1980	2008–2010	2017–2019
Total population difference	15.8	20.6	28.1	20.6	23.2
Effect of factors					
Income decile	24.6	10.2	10.7	4.9	4.7
Occupational category	15.0	7.7	6.5	4.2	3.6
Employment/institution	7.7	5.9	6.6	3.4	2.1
Age	−0.7	3.3	−1.1	−0.3	−0.6
Total composition component	46.7	27.1	22.6	12.1	9.7
Rate component	−30.8	−6.5	5.5	8.5	13.5
B. Percentage difference in marriage rates					
	1960	1970	1980	2008–2010	2017–2019
Total population difference	100.0	100.0	100.0	100.0	100.0
Effect of factors					
Income decile	155.6	49.5	38.0	23.7	20.2
Occupational category	95.1	37.3	23.0	20.2	15.4
Employment/institution	48.4	28.6	23.4	16.4	8.8
Age	−4.2	16.1	−3.9	−1.6	−2.5
Total composition component	294.8	131.5	80.6	58.7	42.0
Rate component	−194.8	−31.5	19.4	41.3	58.0
C. Effect of factors as a percentage of composition component					
	1960	1970	1980	2008–2010	2017–2019
Effect of factors					
Income decile	52.8	37.6	47.1	40.4	48.1
Occupational category	32.2	28.3	28.6	34.4	36.7
Employment/institution	16.4	21.8	29.1	27.9	21.1
Age	−1.4	12.3	−4.8	−2.6	−5.9
Total composition component	100.0	100.0	100.0	100.0	100.0

## Footnotes

2. ACS data are available for 2020, but the Census Bureau has cautioned that because of poor response to the survey during the COVID-19 pandemic, it may not be comparable with prior survey years. US Census Bureau (2021).

3. Heaping at round numbers makes it impossible to divide the population into exact deciles. Moreover, in 2008– 2010 and 2017–2019, men with zero income comprised more than 14% of the population, so the bottom decile is large and the second decile is correspondingly small.

4. In addition to these variables, I experimented with a variety of other socioeconomic factors, including educational attainment, educational enrollment, hours worked in the last week, weeks worked in the last year, and class of worker. I also experimented with controls for region, metropolitan residence, interstate migration, and migration from the South. None of these indicators contribute appreciably to understanding race differences in marriage rates once the three economic measures described above are controlled.

5. The analysis was conducted with DECOMP version 2.0, open-source software available through the Open Science Foundation, https://osf.io/yzr6k

## References

[R1] Bloome D and Ang S (2020). Marriage and union formation in the United States: Recent trends across racial groups and economic backgrounds. Demography 57: 1753–1786. doi:10.1007/s13524-020-00910-7. 32914334 PMC7907839

[R2] Brien M (1997). Racial differences in marriage and the role of marriage markets. Journal of Human Resources 32: 741–778. doi:10.2307/146427.

[R3] Charles KK and Luoh MC (2010). Male incarceration, the marriage market, and female outcomes. The Review of Economics and Statistics 92: 614–627. doi:10.1162/REST_a_00022.

[R4] Cohen PN, and Pepin JR (2018). Unequal marriage markets: Sex ratios and first marriage among Black and White women. Socius: Sociological Research for a Dynamic World 4: 1–10. doi:10.1177/2378023118791084.

[R5] Cragie TL, Myers SL, and Darity WA (2018). Racial differences in the effect of marriageable males on female family headship. Journal of Demographic Economics 84(3): 231–256. doi:10.1017/dem.2018.3. 30221008 PMC6136656

[R6] Darity WA, Myers SL, and Bowman PJ (1995). Family structure and the marginalization of Black men: Policy implications. In: Tucker MB and Mitchell-Kernan C (eds.). The decline in marriage among African Americans: Causes, consequences, and policy implications. New York: Russell Sage: 263–308.

[R7] Das Gupta P (1978). A general method of decomposing a difference between two rates into several components. Demography 15(1): 99–111. doi:10.2307/2060493. 631402

[R8] Das Gupta P (1992). Standardization and decomposition of rates: A user’s manual. (Bureau of the Census, Current Population Reports Special Studies, Series P23–186). Washington, DC: U.S. Government Printing Office.

[R9] Elliott DB, Simmons T, and Lewis JM (2010). Evaluation of the marital events items on the ACS. US Census Technical and Analytic Reports on the American Community Survey. https://www.census.gov/library/working-papers/2010/demo/elliott-08.html.

[R10] Fitch CA and Ruggles S (2000). Historical trends in marriage formation. In: Waite L and Bachrach C (eds.). Ties that bind: Perspectives on marriage and cohabitation. Hawthorne: Aldine de Gruyter: 59–88.

[R11] Fossett MA and Kiecolt KJ (1993). Mate availability and family structure among African Americans in U.S. metropolitan areas. Journal of Marriage and the Family 55(2): 288–302. doi:10.2307/352802.

[R12] Kitagawa EM (1955). Components of a difference between two rates. Journal of the American Statistical Association 50(272): 1168–1194. doi:10.1080/01621459.1955.10501299.

[R13] Kuo JCL and Raley RK (2016). Is it all about money? Work characteristics and women’s and men’s marriage formation in early adulthood. Journal of Family Issues 37(8): 1046–1073. doi:10.1177/0192513X14530973. 27158176 PMC4856051

[R14] Lichter DT, LeClere FB, and McLaughlin DK (1991). Local marriage markets and the marital behavior of Black and White women. American Journal of Sociology 96(4): 843–867. doi:10.1086/229610.

[R15] Lichter DT, McLaughlin DK, Kephart G, and Landry DJ (1992). Race and the retreat from marriage: A shortage of marriageable men? American Sociological Review 57(6): 781–799. doi:10.2307/2096123.

[R16] Lloyd K and South S (1996). Contextual influences on young men’s transition to first marriage. Social Forces 74(3): 1097–1119. doi:10.2307/2580394.

[R17] McLaughlin D and Lichter D (1997). Poverty and the marital behavior of young women. Journal of Marriage and Family 59(3): 582–594. doi:10.2307/353947.

[R18] National Center for Health Statistics (1964). Vital statistics of the United States 1960. Volume III – Marriage and Divorce. Washington, D.C.

[R19] National Center for Health Statistics (1974). Vital statistics of the United States 1970. Volume III – Marriage and Divorce. Rockville, MD.

[R20] National Center for Health Statistics (1985). Vital statistics of the United States 1980. Volume III – Marriage and Divorce. Hyattsville, MD.

[R21] Oppenheimer VK, Kalmijn M, and Lim N (1997). Men’s career development and marriage timing during a period of rising inequality. Demography 34(3): 311–330. doi:10.2307/3038286. 9275242

[R22] Raley RK (1996). A shortage of marriageable men? A note on the role of cohabitation in Black–White differences in marriage rates. American Sociological Review 61(6): 973–983. doi:10.2307/2096303.

[R23] Raley RL, Sweeney MM, and Wondra D (2015). The growing racial and ethnic divide in U.S. marriage patterns. The Future of Children 25(2): 89–109. doi:10.1353/foc.2015.0014. 27134512 PMC4850739

[R24] Ruggles S (2015). Patriarchy, power, and pay: The transformation of American families, 1800–2015. Demography 52(6): 1797–1823. doi:10.1007/s13524-015-0440-z. 26511502 PMC5068828

[R25] Ruggles S (2016). Marriage, family systems, and economic opportunity in the United States since 1850. In: McHale SM, King V, Van Hook J, and Booth A (eds.). Gender and couple relationships. Heidelberg: Springer: 3–41. doi:10.1007/9783-319-21635-5_1.

[R26] Ruggles S, Flood S, Foster S, Goeken R, Pacas J, Schouweiler M, and Sobek M (2021). IPUMS USA: Version 11.0 [dataset]. Minneapolis, MN: IPUMS. doi:10.18128/D010.V11.0.

[R27] South SJ and Lloyd KM (1992). Marriage opportunities and family formation: Further implications of imbalanced sex ratios. Journal of Marriage and Family 54(2): 440–451. doi:10.2307/353075.

[R28] Sweeney MM (2002). Two decades of family change: The shifting economic foundations of marriage. American Sociological Review 67(1): 132–147. doi:10.2307/3088937.

[R29] Sweeney MM (2016). Socioeconomic standing and variability in marriage timing in the twentieth century. Annals of the American Academy of Political and Social Science 633(1): 271–291. doi:10.1177/0002716215596975.

[R30] Testa M and Krogh MS (1995). The effect of employment on marriage among Black males in inner-city Chicago. In: Tucker MB and Mitchell-Kernan C (eds.). The decline in marriage among African Americans. New York: Russell Sage Foundation: 59–95.

[R31] Wilson WJ (1987). The truly disadvantaged: the inner city, the underclass, and public policy. Chicago: University of Chicago.

[R32] Wilson WJ and Neckerman KM (1986). Poverty and family structure: The widening gap between evidence and public policy issues. In: Danziger SH and Wienberg DH (eds.). Fighting poverty: What works and what doesn’t. Cambridge, MA: Harvard University Press: 232–259.

[R33] Wood RG (1995). Marriage rates and marriageable men: A test of the Wilson Hypothesis. Journal of Human Resources 30(1): 163–193. doi:10.2307/146195.

[R34] U.S. Census Bureau (2021). An assessment of the COVID-19 pandemic’s impact on the 2020 ACS 1-Year data. ACS Research and Evaluation Report Memorandum Series ACS21-RER-04. https://usa.ipums.org/usa/resources/Assessment_Covid19_impact_2020_one_year.pdf.

